# Amygdalin-folic acid-nanoparticles inhibit the proliferation of breast cancer and enhance the effect of radiotherapy through the modulation of tumor-promoting factors/ immunosuppressive modulators in vitro

**DOI:** 10.1186/s12906-023-03986-x

**Published:** 2023-05-20

**Authors:** Mostafa A. Askar, Gharieb S. El-Sayyad, Mona S. Guida, Eman Khalifa, El Shaimaa Shabana, Ibrahim Y. Abdelrahman

**Affiliations:** 1 Radiation Biology Department, National Centre for Radiation Research and Technology (NCRRT), Egyptian Atomic Energy Authority (EAEA), Cairo, 11787 Egypt; 2 Drug Microbiology Lab, Drug Radiation Research Department, National Center for Radiation Research and Technology, (NCRRT), Egyptian Atomic Energy Authority (EAEA), Cairo, 11787 Egypt; 3 Unit of Genetics, University Pediatrics Hospital, Faculty of Medicine, Mansoura University, Mansoura, 35516 Egypt; 4 Oral Biology Department, Faculty of Oral & Dental Medicine, Delta University for Science and Technology, Mansoura, 11152 Egypt

**Keywords:** *Amygdalin*, Anticancer, Immunosuppressive modulators, Nanoparticles, Radio-sensitization, Tumor promoting factors

## Abstract

**Introduction:**

Breast cancer (BC) cells often develop multiple mechanisms of chemo- and radio-resistance during tumor progression, which is the major reason for the failure of breast cancer therapy. Targeted nanomedicines have tremendous therapeutic potential in BC treatment over their free drug counterparts. Searching for chemo- and radio-sensitizers to overcome such resistance is therefore urgently required. The goal of this study is to evaluate and compare the radio-sensitizer efficacy of *amygdalin*-folic acid nanoparticles (Amy-F) on MCF-7 and MDA-MB-231 cells.

**Materials and methods:**

The effects of Amy-F on MCF-7 and MDA-MB-231 cell proliferation and IC50 were assessed using MTT assay. The expression of proteins involved in several mechanisms induced by Amy-F in MCF-7 and MDA-MB-231 cells, including growth inhibition, apoptosis, tumor growth regulators, immuno-modulators, and radio-sensitizing activities were evaluated via flow cytometry and ELISA assay.

**Results:**

Nanoparticles demonstrated sustained Amy-F release properties and apparent selectivity towards BC cells. Cell-based assays revealed that Amy-F markedly suppresses cancer cell growth and improves radiotherapy (RT) through inducing cell cycle arrest (G1 and sub-G1), and increases apoptosis as well as reduces the proliferation of BC by down-regulating mitogen-activated protein kinases (MAPK/P38), iron level (Fe), nitric oxide (NO), and up-regulating the reactive oxygen species level (ROS). Amy-F has also been shown to suppress the expression of the cluster of differentiation (CD4 and CD80), and interfere with the Transforming growth factor beta (TGF- β)/Interferon-gamma (INF-g)/Interleukin-2 (IL-2)/Interleukin-6 (IL-6)/Vascular endothelial growth factor (VEGF) induced suppression in its signaling hub, while up-regulating natural killer group 2D receptor (NKG2D) and CD8 expression.

**Conclusions:**

Collectively, the novel Amy-F either alone or in combination with RT abrogated BC proliferation.

## Introduction

Breast cancer (BC) is one of the major threats challenging women’s health all over the world. This threat represents the shared type of malignant tumor in females, with the highest mortality rate [1]. There is a high value of breast cancer incidence all over the world, it reached 2.3 million new cases and represents one breast cancer case from every eight cases of all types of cancers. There are 685 thousand breast cancer women were dying in 2020 representing one case in every six cancer deaths in women [2]. Regarding Egyptian statistics on breast cancer, there is 32.4% breast cancer incidence of total cancer incidence in 2020 [3]. Several regimens and protocols are approved as a treatment for this lethal disease, including chemotherapy, radiotherapy, and surgery in addition to different protocols of combination therapy. Chemotherapy remains the first choice for breast cancer treatment. Nevertheless, the deadly side effects resulting from these cytotoxic chemotherapeutics have posed hindrances along the way [4]. Cancer cells generate an immunosuppressive microenvironment called tumor microenvironment (TME) to regulate tumor growth, promote tumor immune escape, and as a source of tumor-promoting factors (TPFs) [5]. TPFs include growth factors, cytokines, extracellular matrix proteins, and hypoxia-inducible factors, among others, which promote tumor growth, survival, and metastasis [6]. The TME is the networks of cells (such as immune cells, immune cell receptors (CD4, CD8, CD80, and NKG2D), and cancer-associated fibroblasts), promoting factors (i.e.: tumor promotors (MAPK and P38), cytokines (IL-2, IL-6, and INF-γ), growth factors (TGF-β and VEGF), hormones, and signal stimulators (Fe, NO, and ROS), associated with the extracellular matrix and surrounding vasculature that surrounds cancer cells. The formation of TME relies essentially on tumor metabolism and therefore, is characterized by its high acidity and hypoxic state [5]. Nanoparticle-mediated drug delivery systems (DDS) have emerged as a promising tool in this direction, as they can be utilized for the treatment of various diseases by circumventing healthy body tissues, thus causing minimal cytotoxicity and cell death in healthy tissues while targeting only diseased tissues [7]. Many nanoparticles have anticancer properties on their own, whereas others are best described as nanocarriers used for ferrying the hydrophobic drugs selectively to the site of neoplasia [8].

Nowadays, reliance on natural products as a source of new drugs represents the core of new research in the field of drug discovery. *Amygdalin*, a naturally occurring vitamin B17 that could be found in the seeds of many plants in the Prunus Rosacea family (apricots, apples, bitter almonds, black cherries, plums, and peaches), is one of those natural products that have received great attention [9]. *Amygdalin* has many great properties, especially its anticancer activity. Mechanistically, *amygdalin* function as an anticancer agent by inducing apoptosis and cell cycle arrest by releasing toxic hydrogen cyanide (HCN) only after its hydrolysis and thereby destroying cancer cells. Unfortunately, the cytotoxic HCN can also permeate normal cells which result in cyanide poisoning and eventual cell damage [9, 10].

Folic acid (FA), improves the targeting efficiency of cancer therapy due to its preferential binding to the folate receptor-α (FR-α) overexpressed on cancer cells. FR-α receptors are known to be over-expressed in BC cells and to have a high affinity for FA, which are typically captured to feed the fast-dividing BC cells [11]. Utilizing FA in therapeutic formulations has many advantages as FA could be considered as a stipulated nutrient that has good stability, and biocompatibility, as well as its biodegradability by tumor microenvironment (TME) allows for the release/availability of anticancer drugs at the tumor site [12]. Drugs encapsulated in nanocarriers are promising therapeutic modalities because they offer drug accumulation potential in tumor tissues thanks to the enhanced permeability and retention (EPR) effects of their nanocarriers, which in turn augments cancer therapeutic efficacy [13]. Based on the aforementioned advantages of FA-functionalized nanocarriers, we have developed a stimuli-responsive FA-functionalized nanoparticle system consisting of *amygdalin* for targeted BC therapy.

Here, we report the synthesis, characterization, physiochemical properties, and cytotoxic profile of Amy-F nanoparticles against BC cells. We extended our goal to gain insight into the effect of novel Amy-F nanoparticles on tumor regulatory mediators’ role through the modulation of tumor promoting factors/ immunosuppressive modulators to restrain BC promotion in vitro.

## Materials and methods

### Synthesis of Amy-F nanoparticles

**Fig. 1:**
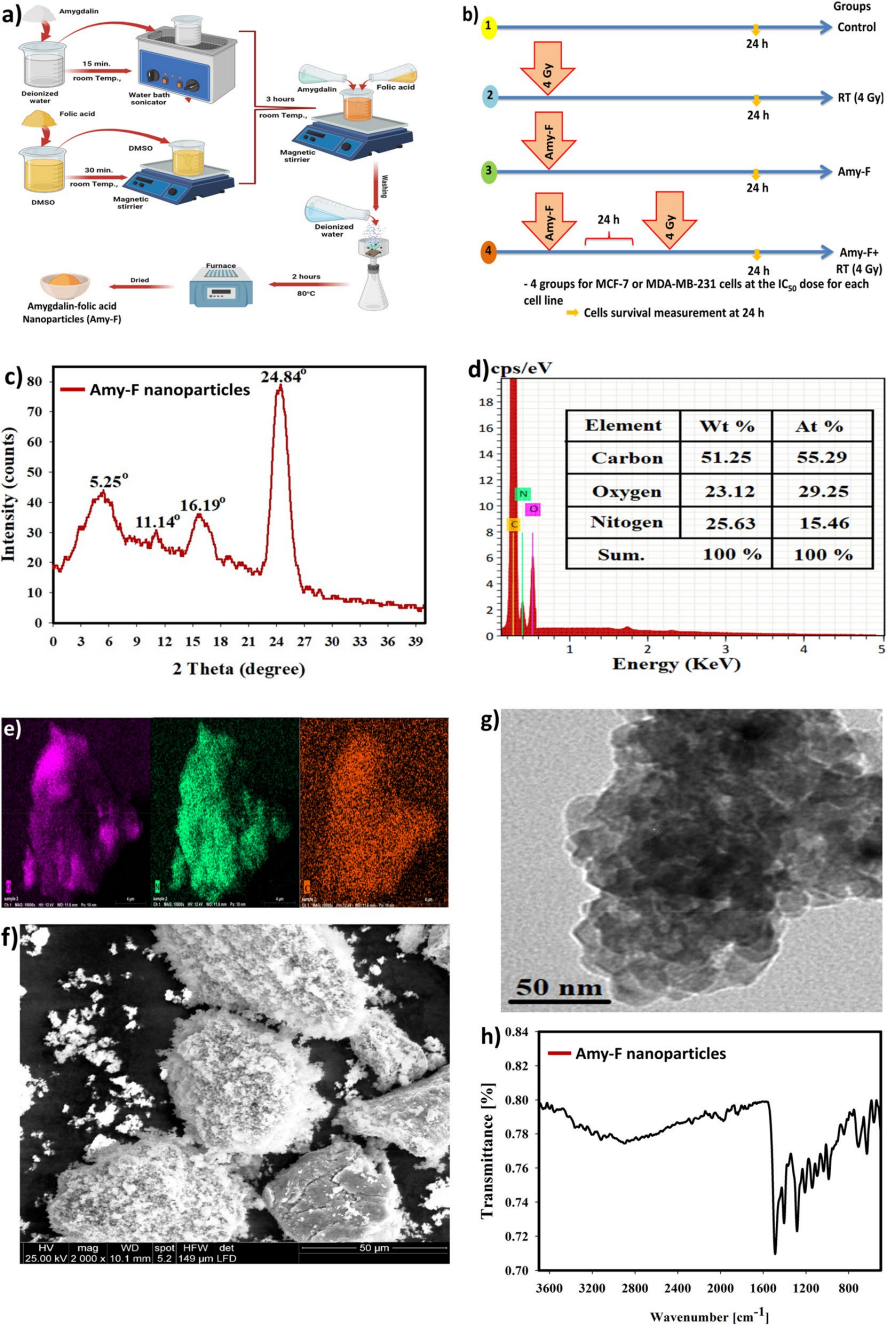
Structural characteristics of Amy-F nanoparticles, and experimental procedures diagram. **a** Diagram representing steps of Amy-F synthesis; **b** Schematic diagram illustrated the experimental procedures; **c** XRD diffraction pattern of Amy-F nanoparticles; **d** EDX elemental analysis of the synthesized Amy-f nanoparticles; **e** EDX elemental mapping of Amy-F nanoparticles; **f** SEM image of the synthesized Amy-F; **g** HR-TEM image of the synthesized Amy-F; **h** FTIR analysis of the synthesized Amy-F nanoparticles

The *amygdalin* solution was sonicated at 25 °C for 15 min to form an aqueous solution (10 mM) of *amygdalin* (vitamin B17). In a separate glass vial, 25 mg of folic acid was dissolved in 25 mL of dimethyl sulfoxide (DMSO) while stirring. The folic acid solution was then added to the *amygdalin* solution under vigorous stirring for 3 h. The resultant solution was then washed using MilliQ water and allowed to dry in an oven at 80 °C. The resultant *amygdalin*-folic acid nanoparticles (Amy-F NPs) were stored at room temperature for further work (Fig. 1a).

### Characterization of Amy-F nanoparticle

High-resolution transmission electron microscopy (HR-TEM) (JEOL JSM-5600 LV, Japan) was used to obtain the size and shape of the synthesized Amy-F nanoparticles. Using the X-ray diffraction technique (XRD; Shimadzu XRD-6000) the amorphous structure of the synthesized Amy-F nanoparticle samples was inspected. XRD spectra were acquired in the range of 2θ from 17° to 90° at 25 °C. Copper K-α is a radiation source of scan rate 0.8°/min, wavelength λ = 0.15408 nm, current 40 mA and operation voltage 50 kV. Information about the surface morphology and appearance of the samples' particles is obtained using scanning electron microscopy (SEM), JEOL JSM-5600 LV, Japan). FTIR analysis (JASCO FT-IR 3600, KBr Pellet method, and wavenumber range from 400 to 4000 cm^−1^) was carried out to reveal chemical functional groups established among the prepared sample. Finally, the energy dispersive X-ray spectra (EDX) (JEOL JSM-5600 LV, Japan) were examined to confirm nanoparticle formation.

### Cell lines and culture media

Breast cancer cell lines (MCF-7 and MDA-MB-231) and normal cell lines (MCF-10A) were purchased from the cell culture department, VACSERA, Cairo, Egypt. The cells were cultivated by the distributor's instructions in high glucose RPMI 1640 medium (Thermo Fisher Scientific Inc., USA) in addition to 10% FBS (Thermo Fisher Scientific Inc., USA), penicillin (100 U/mL) and streptomycin (100 μg/mL) and incubated at 37 °C in a humid environment (5% CO_2_) [14].

### Cytotoxicity assay

Cellular viability and morphological assay were analyzed to indicate the cytotoxic profile of Amy-F nanoparticles using the 3-(4, 5-Dimethylthiazol-2yl)-2, 5-diphenyl tetrazolium bromide (MTT) (Sigma-Aldrich, USA) assay according to Van-de et al. [15]. Amy-F (100 µg/mL) was dissolved in propylene glycol (Sigma-Aldrich, USA). Before use, dilution of the Amy-F stock solution was carried out to the indicated concentration using a culture medium, and the final concentration of propylene glycol was 0.1% (v/v) in each well. The control wells contain cells that received the vehicle (propylene glycol) treatment only. In a tissue culture plate (96-well), the wells were inoculated with 1 X 10^5^ cells/mL (100 µL/well) followed by incubation for 24 h at 37ºC to generate a complete monolayer sheet. Following the formation of a confluent cell sheet, decantation of the growth medium was carried out, and washing of the cell monolayer was performed twice using washing media. Right after that, the Amy-F was diluted twice in RPMI medium enriched with 2% maintenance serum medium. A volume of 0.1 mL of each dilution was then examined in different wells, with three control wells that contain only maintenance medium. After incubation, the plate was examined under the microscope for any signs of physical toxicity (cell shrinkage, rounding or shrinkage, monolayer loss entirely or partially). An MTT solution (5 mg/ml in PBS) was prepared, and each well received 20 µL of the solution. The plate was then placed on a plate shaker (150 rpm/5 min) to ensure the homogeneous distribution of MTT into the medium. The cells were then incubated at 37ºC and 5% CO_2_ for 5 h to ensure MTT metabolization. Each well was then treated with 200 µL of DMSO after removing the media to solubilize the resultant formazan crystals (MTT metabolic product). The absorbance of each well was then recorded at 570 nm using an ELISA plate reader (BioTeck, Bad Friedrichshall, Germany). Using SPSS (IBM Inc., USA) one-way ANOVA, the half-maximum inhibitory concentration (IC50) was calculated. Graph-Pad Prism software (v.8.0) (Graph-Pad Prism Inc., USA) was used to create the graphs. Cell morphology was recorded using an inverted microscope with a digital camera (Nikon, Japan). All studies were carried out in triplicate.

### In vitro Amy-F release

The in vitro drug release study was carried out as demonstrated by Askar et al. [14]. At 37 °C, the Amy-F suspensions were subjected to different pH at 6, 7, and 9. After 24 h of incubation, under UV irradiation using a 350 W mercury-vapour lamp endowed with an optical filter UG5 that allows selecting the 200–380 nm spectral range, where there are the most intense Hg spectral lines at 253 nm. The FA release in phosphate buffer PB solutions was measured using Lambda 950 model UV–VIS-NIR spectrophotometer (Perkin Elmer) [16].

### Cellular selectivity and Amy-F uptake

Normal (MCF-10) and BC cells (MCF-7 and MDA-MB-231) were sowed at 2 × 10^4^ cells/well density in 24-well plates using round coverslips. The cells were incubated with 100 µg/mL Amy-F for 24 h. Following incubation for 24 h, the cells were washed in phosphate buffer solution (PBS) three times and divided into two aliquots for divergent methods; the first method was used to investigate Amy-F cellular selectivity according to Askar et al. [15] by determining the expression of FR-α expression via qRT-PCR, as demonstrated later in the real-time PCR part.

The second method was to employ a UV–VIS-NIR spectrophotometer in estimating folic acid (FA) concentration in normal and cancer cells as well as through uptake quantity of FA that reflects the Amy-F uptake in normal and BC cells. Before use, a standard solution of FA was prepared by serially diluting a 1000 mg/L stock solution (Scharlau Chemie, Barcelona, Spain). After 24 h, the cells were rinsed three times with PBS, centrifuged at 3000 rpm for 15 min, and the supernatant was aspirated into another plain bottle using a Pasteur pipette. Supernatant and pellet cell samples were diluted with MilliQ water and homogenized before the determination of FA concentration using Lambda 950 model UV–VIS-NIR spectrophotometer (Perkin Elmer) at 200–380 nm spectral range.

### Radiation facility

All the MCF-7 and MDA-MB-231 cells that underwent irradiation were irradiated with gamma rays (γ-rays) at 85% confluency as a single shot (single exposure to 4 Gy) at a dose rate of 0.012 Gy/Sec using a ^137^Cs source (Gamma-cell-40 Exactor; NCRRT, EAEA, Cairo, Egypt). The dosimetry was applied in all experiments to ensure dose uniformity and dose rate employing a Fricke reference standard dosimeter [15].

### Cell culture models and the study protocol

Amy-F’s anti-proliferative and radiosensitizing effectiveness was studied by dividing MCF-7, and MDA-MB-231 cell cultures into four distinct groups as follows, described in (Fig. 1b):

MCF-7 cell line.

I) MCF-7 cell line: MCF-7 group: untreated MCF-7cells served as control
II) MCF-7 + Amy-F (Amy-F group): MCF-7 cells treated with Amy-F
III) MCF-7 + RT (4 Gy) (RT group): MCF-7 cells exposed to single γ-rays (4 Gy)
IV) MCF-7 + Amy-F + RT (Amy-F + RT group): MCF-7 cells treated with Amy-F and exposed to single γ-rays (4 G).

MDA-MB-231 cell line.

I) MDA-MB-231 cell line: MCF-7 group: untreated MDA-MB-231cells served as control
II) MDA-MB-231 + Amy-F (Amy-F group): MDA-MB-231 cells treated with Amy-F
III) MDA-MB-231 + RT (4 Gy) (RT group): MDA-MB-231 cells exposed to single γ-rays (4 Gy)
IV) MDA-MB-231 + Amy-F + RT (Amy-F + RT group): MDA-MB-231 cells treated with Amy-F and exposed to single γ-rays (4 G).

After 24 h of incubation following the radiotherapy dose, cells were harvested for further investigations to reveal Amy-F’s antitumor effect.

### Cell cycle, apoptosis, CD4, CD8, CD80, and TGF-β analysis by flow cytometry

After 24 h of incubation following the last dose of radiotherapy, the 3 × 10^5^ cells/well were harvested with trypsin, washed twice in ice-cold PBS, and then fixed with 70% ethanol at 4 °C overnight for all groups of both MCF-7 and MDA-MB-231 cell lines. Afterwards, the cells were washed with PBS and centrifuged before being stained with propidium-iodide (PI) (50 µg/mL) for cell cycle analysis (Cat. No: ab139418) and apoptosis markers (BCl-2, Caspase-3, CD4, CD8, CD80, and TGF-β) were measured using FITC Kits (Cat. No: 340575, 550,480, 557,767, 557,766, 567,442, and 562,962, respectively; Beckman Coulter, Marseille, France). The staining was evaluated using a FACSCanto-II flow cytometer, and the data were analyzed using BD Accuri-C6 Plus software (Biosciences, CA, USA) [17].

### Determination of MAPK, P38, Fe, and ROS

The levels of phospho-MAPK (Ser-93), p38 (Phospho-Tyr-323), Fe, and intracellular ROS in treated and vehicle-treated MCF-7 and MDA-MB-231 cells were measured. Cells were seeded in a 6-well plate (2 × 10^6^ cells/well) overnight in a complete medium, then harvested and homogenized in 100 µL ice-cold water. The levels of MAPK, P38, Fe, and ROS were measured using MyBioSource ELISA kits, Cat.No: MBS629151, MBS9404731, MBS267375, and MBS039665, respectively (MyBiosource, Inc. Southern California, San Diego, USA). Reactions were carried out following the manufacturer's protocol and absorbance was determined using an automatic microplate reader (Quant, BioTek Instruments, Inc., Winooski, VT, USA).

### RNA isolation and real-time qRT- PCR analysis

Total RNA was extracted from MCF-7 and MDA-MB-231 cells utilizing Trizol Reagent (Thermo Fisher Scientific). The PrimeScriptTM RT reagent kit was then used to extract cDNA from total RNA (Takara Bio Inc., Otsu, Japan). Quantitative reverse transcription polymerase chain reaction (qRT-PCR) was used to estimate the mRNA expression, which was done in triplicate utilizing an SYBR Premix Ex Taq TM kit (Takara Bio, Inc.) and an ABI 7900HT Real-Time PCR system (Thermo Fisher Scientific). The primer sequence is as follows: for Homo sapiens folate receptor alpha, transcript variant 7, mRNA (FR-α) expression: forward primer; 5’-CTGGCTGGTGTTGGTAGAACAG-3’ and reverse primer; 5’-AGGCCCCGAGGACAAGTT-3’ (Genecode: NM_016724.3); and for Homo sapiens glyceraldehyde-3-phosphate dehydrogenase (GAPDH), transcript variant 6 as a housekeeping gene: forward primer; 5’-GTCAAGGCTGAGAACGGGAA-3’ and reverse primer; 5’-AAATGAGCCCCAGCCTTCTC-3 ( Gene code: NR_152150.2)’. The comparative cycle threshold values (2– ΔΔCt) [18] were applied to assess the final results, as the GAPDH gene expression was utilized to normalize qRT-PCR results.

## Statistical analysis

All experiments were carried out in triplicate, and the results were stated as the mean ± standard error (SEM). The Kolmogorov–Smirnov (KS, *p* > 0.10) test verified data normality, and all data were found to be normally distributed. ANOVA and Tukey multiple comparisons post hoc tests was used for data analysis. Statistical analyses were carried out using Prism, version 8 (GraphPad Software, La Jolla, CA). The *p* < 0.001, *p* < 0.01, and *p* < 0.05 statistical significance levels were applied to indicate the difference between groups.

## Results

### Structural characterization of Amy-F nanoparticles

As illustrated in the XRD diffractogram, the synthesized Amy-F nanoparticle revealed the amorphous nature of the organic vitamin B17 and folic acid (Fig. 1c). The generated XRD models agree with the original standard card for both *amygdalin* and folic acid. The following two unique peaks are located at 11.14°, and 24.84° corresponding to folic acid amorphous structure [19]. There are other important peaks noted at 2Ɵ = 5.25°, and 16.19°, which corresponding to the *amygdalin* XRD pattern [20]. The most significant diffraction peaks were slightly changed due to the new construction of the Amy-F form.

EDX spectrum of Amy-F nanoparticles showed the coexistence of O, C, and N which are accredited to the organic nature of both *amygdalin* and FA [15] (Fig. 1d). EDX elemental mapping revealed the uniform distribution of O, C, and N throughout the Amy-F nanoparticles (Fig. 1e).

The SEM analysis showed an irregular structure of the synthesized Amy-F nanoparticles with remarkable smooth agglomerates (Fig. 1f) due to the occupation of a large number of layers at the grain boundary, which could control grain growth [21]. Furthermore, the HR-TEM image confirms the irregular structure of Amy-F nanoparticles with diameters ranging from 80—215 nm and an average diameter of 155.58 nm (Fig. 1g).

FT-IR spectroscopy was carried out to investigate the surface functional groups of the synthesized Amy-F nanoparticles. The FT-IR spectrum of the synthesized Amy-F nanoparticles is shown in Fig. 1h. Concordantly with the literature, the characteristic IR absorption peaks at 1691, 1608, and 1515 cm^−1^ are observed in the FTIR spectrum assigned to folic acid, and are caused by N–H bending vibration of the CONH group, C = O amide stretching of the α-carboxyl group, and absorption band of the phenyl ring, respectively [22]. A band at 3005 cm^−1^ is attributed to the OH and NH stretching regions. The presence of narrow peak bands at 1759 cm^−1^ in *amygdalin* IR spectra is due to aldehyde and ketone C = O stretching [23]. The position of the C = O stretching indicated the hydrogen bonding and conjugation within the molecules [24]. High intensity peaks, followed by peaks at 2900 cm^−1^ and 2773 cm^−1^, are attributed to O–H stretching (carboxylic acid) vibrations and aldehyde C-H stretching. This O–H stretching vibration may be due to carboxylic compounds in the polymer protein matrix. Finally, the absorption band at 1409 cm^−1^, 1325 cm^−1^, and 700 cm^−1^ were assigned to amide II, amide III, and amide IV, respectively [25]. Depending on the comparison achieved between the FTIR data of bare FA [26] and *amygdalin* [27], it is worth mentioning that the connection type between the FA and amygdalin was by intermolecular hydrogen bonding (weak bond) as described previously, which was not present in bare FA and *amygdalin*, that indicated the incorporation behavior between FA and *amygdalin* as demonstrated by a weak bond [28]. In our FTIR results, the incorporation behavior was detected as new peaks formed in the synthesized nanoparticles (weak physical bond; Van der Waals forces) [29, 30]. Figure 1h presented the chemical structure of FA and *amygdalin*, and explained the type of connection between FA and *amygdalin*.

### Cytotoxicity assay

**Fig. 2:**
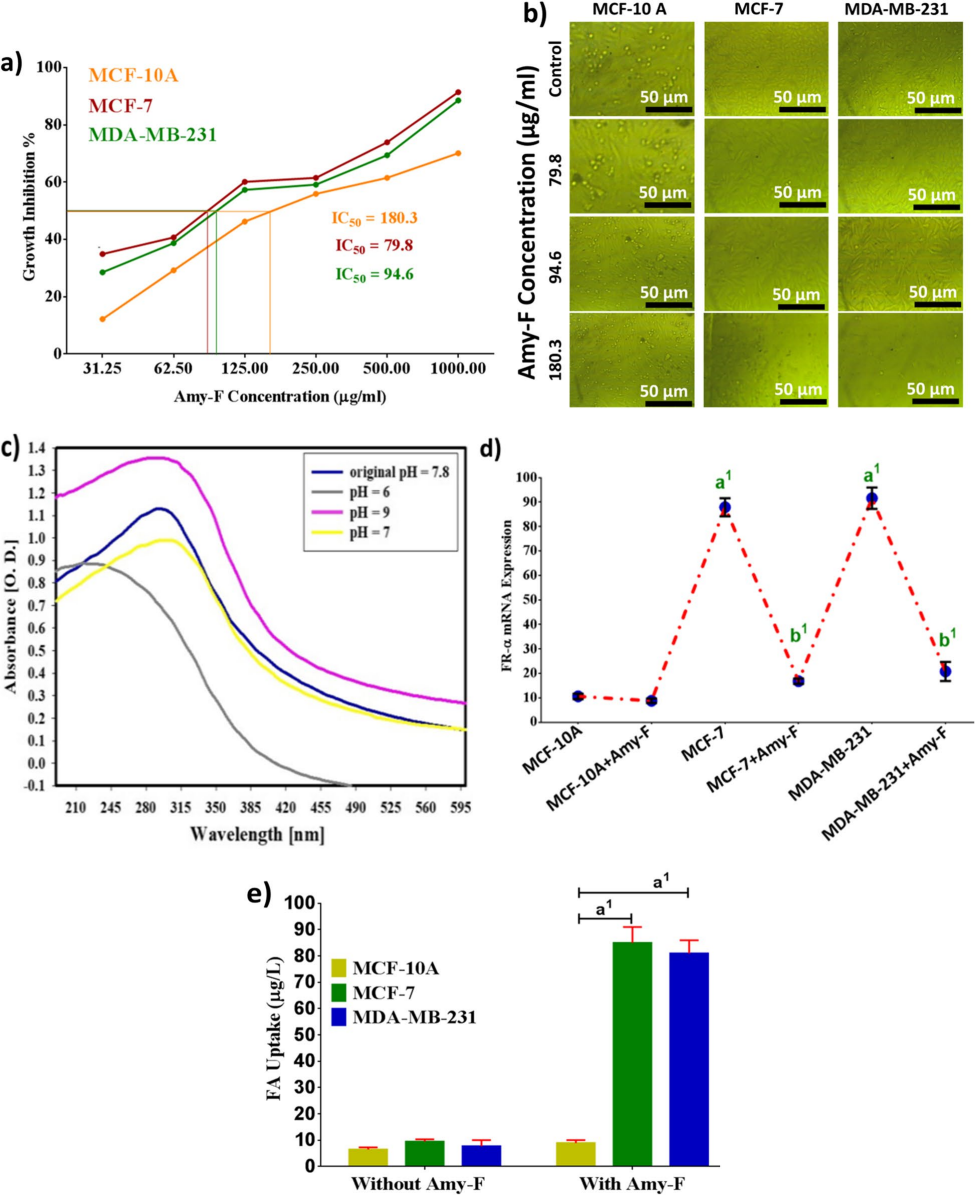
Cytotoxicity and inhibition of BC cells proliferation, in vitro release, enhanced selectivity, and cellular uptake of Amy-F nanoparticles. **a** MTT assay of BC and MCF-10A cells treated with Amy-F at different concentrations; **b** Representative phase-contrast images of MCF-10A, MCF-7, and MDA-MB-231 cells captured by the inverted light microscope; **c** The release of Amy-F at different pH; 6, 7, and 9 dispersed in PBS containing DMSO 0.1% estimated using UV–vis spectrometry; **d** Representative histogram of the FR-α expression in normal (MCF-10A) and BC cells; **e** Intracellular FA uptake values in MCF-10A, MCF-7, and MDA-MB-231 cells after 24 h incubation with Amy-F at IC50 doses. Values are represented as the mean ± standard error of the mean (SEM) of triplicate samples from two independent experiments. Values at a1p < 0.001, a2p < 0.01 vs untreated normal cells; b1p < 0.001, b2p < 0.01 vs. untreated cancer cells are considered significant

As represented in (Fig. 2a), the IC50 of Amy-F on MCF-10A was 180.3 μg/mL. Whereas, the data of the anticancer effect of Amy-F on BCCs (MCF-7 and MDA-MB-231) showed an anti-proliferative activity against the cancer cell lines after 24 h. The IC50 of Amy-F was revealed at 79.8 µg/mL and 94.9 μg/mL for MCF-7 and MDA-MB-231 cells, respectively. Based on the above data, it could be concluded that Amy-F exerts no cytotoxic effect on normal cells. Amy-F concentration-dependent cell morphological alterations were shown in phase-contrast images (Fig. 2b). There was evidence of cell fragmentation, apoptotic cellular shrinkage, membrane blebbing, and disengage characteristics observed in MCF-7 and MDA-MB-231 cells treated with Amy-F nanoparticle compared to the intact MCF-10A-normal cells.

### Amy-F release

The pH-dependent drug-releasing properties of Amy-F were studied in vitro by using UV–Vis at different pH (6, 7 and 9) in phosphate buffer solutions (PBS) containing DMSO 0.1%, to simulate the neutral environment of normal cells and acidic conditions in cancer cells. As shown in (Fig. 2c), at pH 6, there is more than 55% release of Amy-F. Whereas, at pH 7 and 9 the release of Amy-F was less than 10% and 1%, respectively, after 24 h. The elevated release of Amy-F in acidic environments could be ascribed to the protonation and high solubility of Amy-F in such environments.

### Cellular selectivity and uptake of Amy-F

To test the selectivity of Amy-F towards cancer cells, FR-α gene expression was evaluated in MCF-10A cells, MCF-7 and MDA-MB-231. Treating MCF-10A with Amy-F showed a non-significant change in the levels of FR-α (Fig. 2d) compared to untreated MCF-10A cells. On the other hand, a significant elevation in FR-α expression was observed in untreated MCF-7 and MDA-MB-231 (by 8.3 and 8.6 fold, respectively) compared to untreated MCF-10A. Whereas, a significant reduction in the expression of FR-α was observed in MCF-7 and MDA-MB-231 after treatment with Amy-F nanoparticles (by 80.9% and 77.3%, respectively) compared to untreated MCF-7 and MDA-MB-231, respectively. Based on this data, it could be concluded that Amy-F is only selective to FR-α receptors that are over-expressed on breast cancer cell membranes due to FA moiety which is incorporated into the Amy-F nanoformulation.

The data of the UV–VIS-NIR spectrophotometer in (Fig. 2e) show the cellular uptake of Amy-F in normal cells and both cancer cells. Quantitative data showed an elevated uptake of Amy-F by MCF-7 and MDA-MB-231 cells (7.2 and 7.6- fold, respectively) than normal cells. These results confirmed a selective uptake of Amy-F by BCCs compared to normal cells.

In contrast, the uptake of nanoparticles from cancer cells treated with Amy-F was higher with a significant difference at *p* < 0.001. The uptake of Amy-F particles was calculated depending on the intracellular concentration of Amy-F. These values are measured against the estimated number of Amy-F introduced to the MCF-7 and MDA-MB-231 cells, ~ 66.5 and 84.6% of nanoparticles, respectively, from IC50 dose are more efficiently internalized than normal cells.

### Amy-F and/or RT-induced cell cycle arrest and apoptosis in BC cells

To evaluate the role of Amy-F as an anticancer and radio-sensitizer in BCCs, cell cycle distribution and cell apoptosis by flow-cytometry were carried out.

**Fig. 3:**
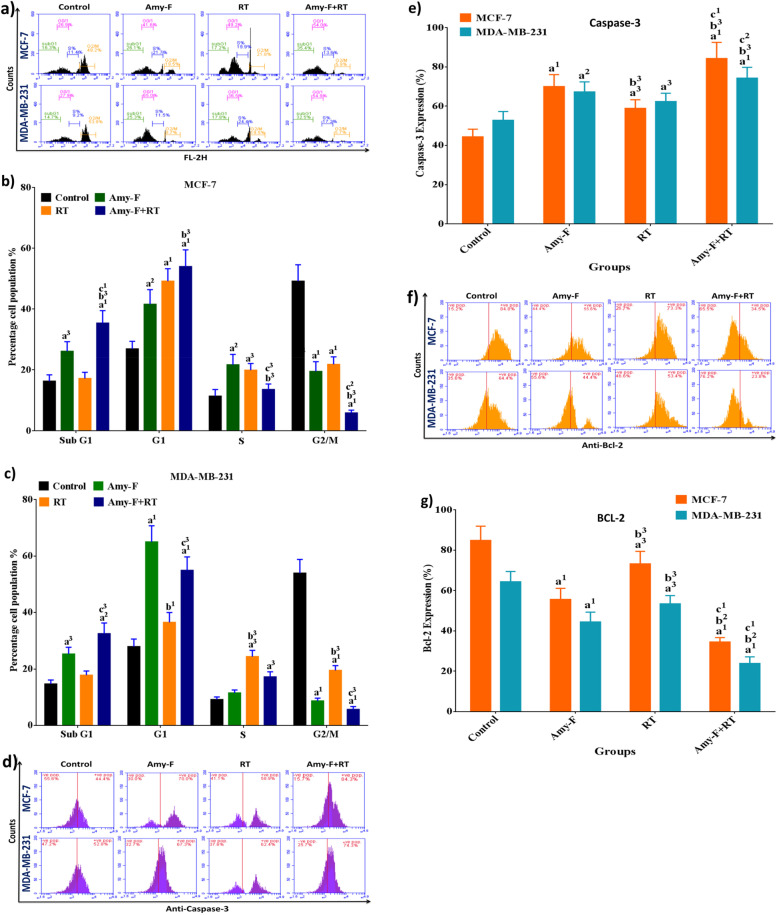
Amy-F and/or RT promote cell cycle arrest and apoptosis: **a**, **b**, and **c** Cell cycle images of MCF7 and MDA-MB-321 cells and histogram represent the data, respectively, **d** and **e** Caspase-3 images of MCF7 and MDA-MB-321 cells and histogram represent the data, respectively, **f** and **g** Bcl-2 images of MCF7 and MDA-MB-321 cells and histogram represent the data, respectively, Data are mean ± SEM, a1p < 0.001, a2p < 0.01, a3p < 0.05 vs. Control group; b1p < 0.001, b2p < 0.01, b3p < 0.05 vs. Amy-F group; c1p < 0.001, c2p < 0.01, c3p < 0.05 vs. RT group

The cell cycle analysis of untreated BCCs showed accumulation in the G2/M phase. After treatment with Amy-F, remarkable elevation in G1 and sub-G1 phases was observed in BCCs when compared to groups of BCCs and 4 Gy of each BCC. Whereas, the cells exposed to 4 Gy displayed arrest at the G1 phase with remarkable elevation in the sub G1 phase compared to the control cells (Fig. 3a,b,c). Furthermore, the combination group Amy-F + 4 Gy revealed a higher proportion in G1 and sub-G1 phases in both cell lines as compared to BCCs, 4 Gy, and Amy-F of each BCC.

The analysis of apoptotic markers showed a significant elevation of caspase-3 (Fig. 3d and e), and a significant decrease in the percentage of Bcl-2 (Fig. 3f and g) in the Amy-F group in both cell lines as compared to BCCs and RT groups, except caspase-3 in MDA cells (Fig. 3d and e). Furthermore, the RT group exhibited a slight elevation in the percentage of caspase-3 and reduction of the Bcl-2 percentage in both cell lines as compared to untreated BCCs groups. Additionally, it was observed that treating BCCs with Amy-F + RT induces an increase in caspase-3 percentage (Fig. 3d and e), and a decrease of Bcl-2 (Fig. 3f and g) compared to BCCs, Amy-F, and RT of each type.

### Modulatory effect of Amy-F and/or RT on tumor promoting factors

**Fig. 4:**
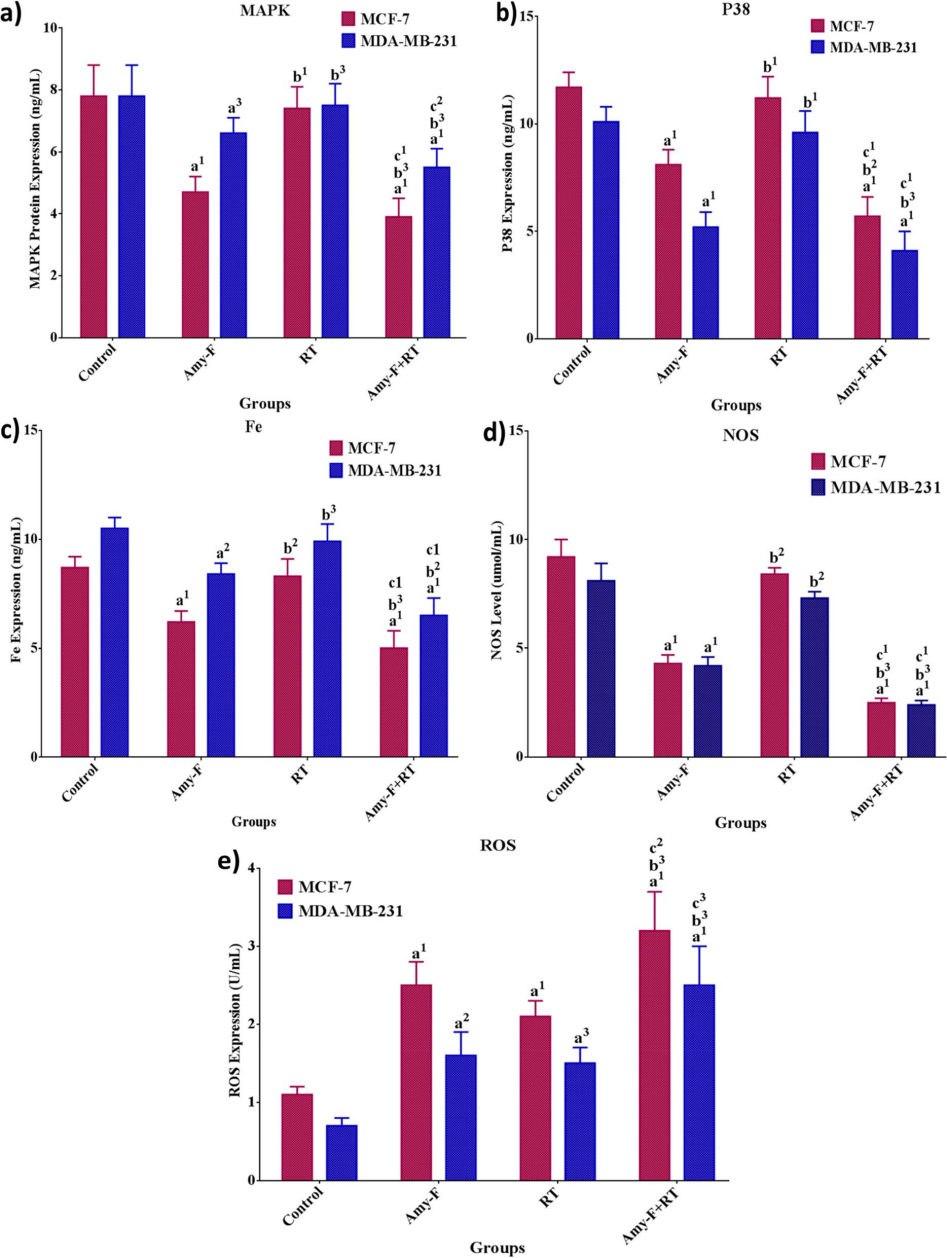
The impact of Amy-F and/or RT on MAPK, P38, Fe, NO, and ROS levels in BC cells. **a** The protein expression of MAPK in MCF-7 and MDA-MB-231 cells, respectively. Protein levels of P38 (**b**), Fe (**c**) in MCF-7 and MDA-MB-231 cells, respectively. The protein expression ratio of NO (**d**) and ROS (**e**) in MCF-7 and MDA-MB-231 cells, respectively. Data are mean ± SEM, a1p < 0.001, a2p < 0.01, a3p < 0.05 vs. Control group; b1p < 0.001, b2p < 0.01, b3p < 0.05 vs. Amy-F group; c1p < 0.001, c2p < 0.01, c3p < 0.05 vs. RT group

To further elaborate on how the combined treatment of BCCs with Amy-F and RT induced anticancer activity and improved the radiosensitivity, we examined the tumor promoting factors expression, as well as the MAPK/P38/Fe/NO/ROS signaling axis. After 24 h, the control and treated cells were harvested and subjected to mediators analysis. As revealed in (Fig. 4 a-e), cells treated with Amy-F showed a significant reduction in MAPK, p38, Fe, and NO expression by (MCF-7; 39.7, 30.7, 28.7, and 53.3%, respectively), and (MDA; 15.4, 48.5, 22.1, and 48.1%, respectively), along with a significant elevation of ROS expression by 2.2 fold for MCF-7 and MDA compared to the control group. Moreover, cells treated with Amy-F induced a significant reduction in the MAPK, p38, Fe, and NO levels by (MCF-7; 36.5, 27.7, 25.3, and 48.8%, respectively), and (MDA; 12, 45.8, 15.2, and 42.5%, respectively) in comparison with the RT group.

Exposure to 4 Gy induced a significant increase in the levels of ROS by twofold for MCF-7 and MDA compared to the control group.

In contrast, the combination of Amy-F + RT elicited a profound reduction in the MAPK, p38, Fe, and NO levels in MCF-7 and MDA-MB-231 cells (~ 50%) as compared to control and RT groups, and (~ 20%) as compared to Amy-F group (Fig. 4 a-e). Furthermore, the ROS levels were significantly elevated by 2 and 2.5 fold for MCF-7 and MDA cells, respectively, compared to the control group. Whereas, the combined treatment with Amy-F + RT results in a significant increase in ROS levels by ~ 1.5 fold for MCF-7 and MDA cells when compared with Amy-F and RT groups, suggesting that the preemptive treatment with Amy-F before RT could hinder the excessive metabolic activity associated with ROS overproduction in both subtypes of BC cells.

### Amy-F and/or RT down-regulated CD4 expression, synergized CD8-mediated suppression of CD80, and activated NKG2D expression in human BC cells

To further elaborate on how the combined treatment of BCCs with Amy-F nanoparticle and RT induced anticancer activity and improved the radio-sensitivity, we examined the CD expression of the key regulatory protein, CD4, CD8, CD80 as well as the NKG2D.

**Fig. 5:**
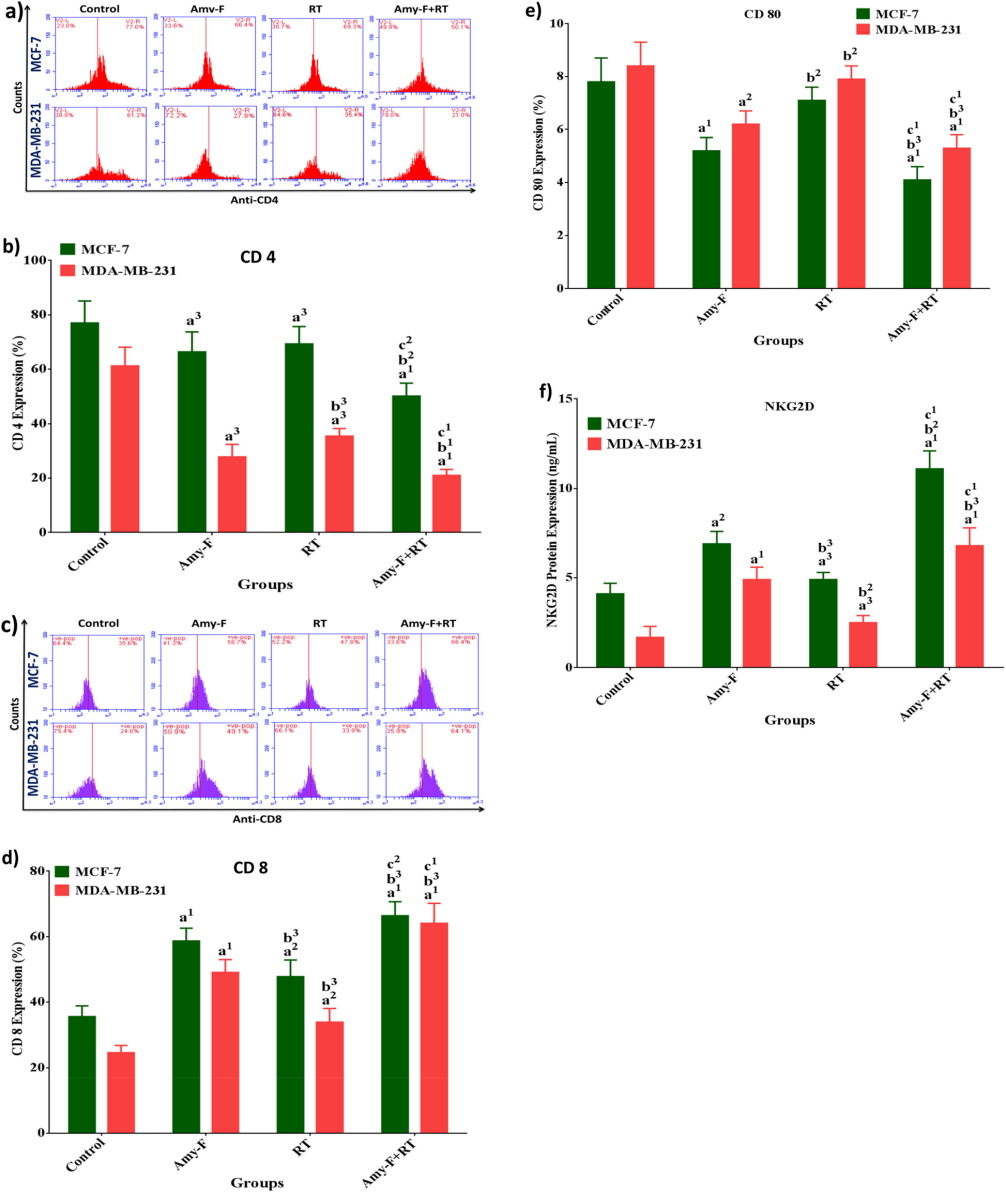
Expression of CD4, CD8, CD80, and NKG2D in BC cells upon treatment with Amy-F and/or exposed to 4 Gy γ-irradiation. **a**, **b** % change in the expression of CD4 in Amy-F and/or RT treated MCF-7 and MDA-MB-231 cells, respectively; **c**, **d** Fold change in the expression ratio of CD8 in Amy-F and/or RT treated MCF-7 and MDA-MB-231 cells, respectively; **e** % change in the expression of CD80 in Amy-F and/or RT treated MCF-7 and MDA-MB-231 cells, respectively; **f** Fold change in the expression ratio of NKG2D in Amy-F and/or RT treated MCF-7 and MDA-MB-231 cells, respectively. Data are mean ± SEM, a1p < 0.001, a2p < 0.01, a3p < 0.05 vs. Control group; b1p < 0.001, b2p < 0.01, b3p < 0.05 vs. Amy-F group; c1p < 0.001, c2p < 0.01, c3p < 0.05 vs. RT group

MCF-7 and MDA-MB-231 cells were treated with Amy-F nanoparticles at IC50 doses for each cell line and/or exposed to 4 Gy (RT). After 24 h, the control and treated cells were harvested and subjected to flow cytometry and ELISA analysis of the target proteins. As revealed from Fig. 5a and b, MCF-7 cells treated with Amy-F and/or RT showed a significant reduction in CD4 expression by 13.7, 10, and 34.9%, respectively, along with a significant reduction in CD4 expression in MDA-MB-231 cells by 54.5, 42.1, and 65.6%, respectively, for Amy-F, RT, and Amy-F + RT groups, respectively, compared to the control group. In turn, this is coupled with a pronounced elevation in CD8 expression ratios (Fig. 5c and d), by 1.6, 1.3, and 1.8 folds, respectively, for Amy-F, RT, and Amy-F + RT groups, respectively in MCF-7 cells, and by 1.9, 1.3, 2.6 folds, respectively, for Amy-F, RT, and AF + RT groups, respectively in MDA-MB-231 cells compared to the control cells. Whereas, a marked reduction in CD80 expression levels was observed by 33.3, and 47.4%, respectively, in MCF-7 cells and by 26.2 and 36.9%, respectively, for Amy-F and AF + RT groups, in MDA-MB-231 cells as compared to control cells (Fig. 5e). More importantly, NKG2D protein expression was augmented by 1.7, 1.1, and 2.7 folds in MCF-7 cells from the Amy-F, RT, and Amy-F + RT groups, respectively, and by 2.9, 1.5, 4 folds in MDA-MB-231 cells from the Amy-F, RT, and Amy-F + RT groups, respectively, compared to the control group (Fig. 5f). Cancer cells treated with Amy-F showed a considerable reduction in the CD4, CD80 expression, whereas, induced elevation in CD8 and NKG2D expression was observed in Amy-F treated BCCs when compared to the RT group. In contrast, the combination of Amy-F + RT results in a profound decrease in the CD4 and CD80 expression, and an increase in the CD8 and NKG2D expression in MCF-7 and MDA cells compared to Amy-F and RT groups, suggesting that the preemptive treatment with Amy-F prior to RT could hinder the excessive metabolic activity associated with immunomodulatory in both subtypes of BC cells.

### Amy-F and/or RT down-regulated TGF-β and blocked VEGF- induced angiogenic tendency through inhibiting INF-γ activation and reducing IL-2/IL-6 levels in human BC cells

**Fig. 6:**
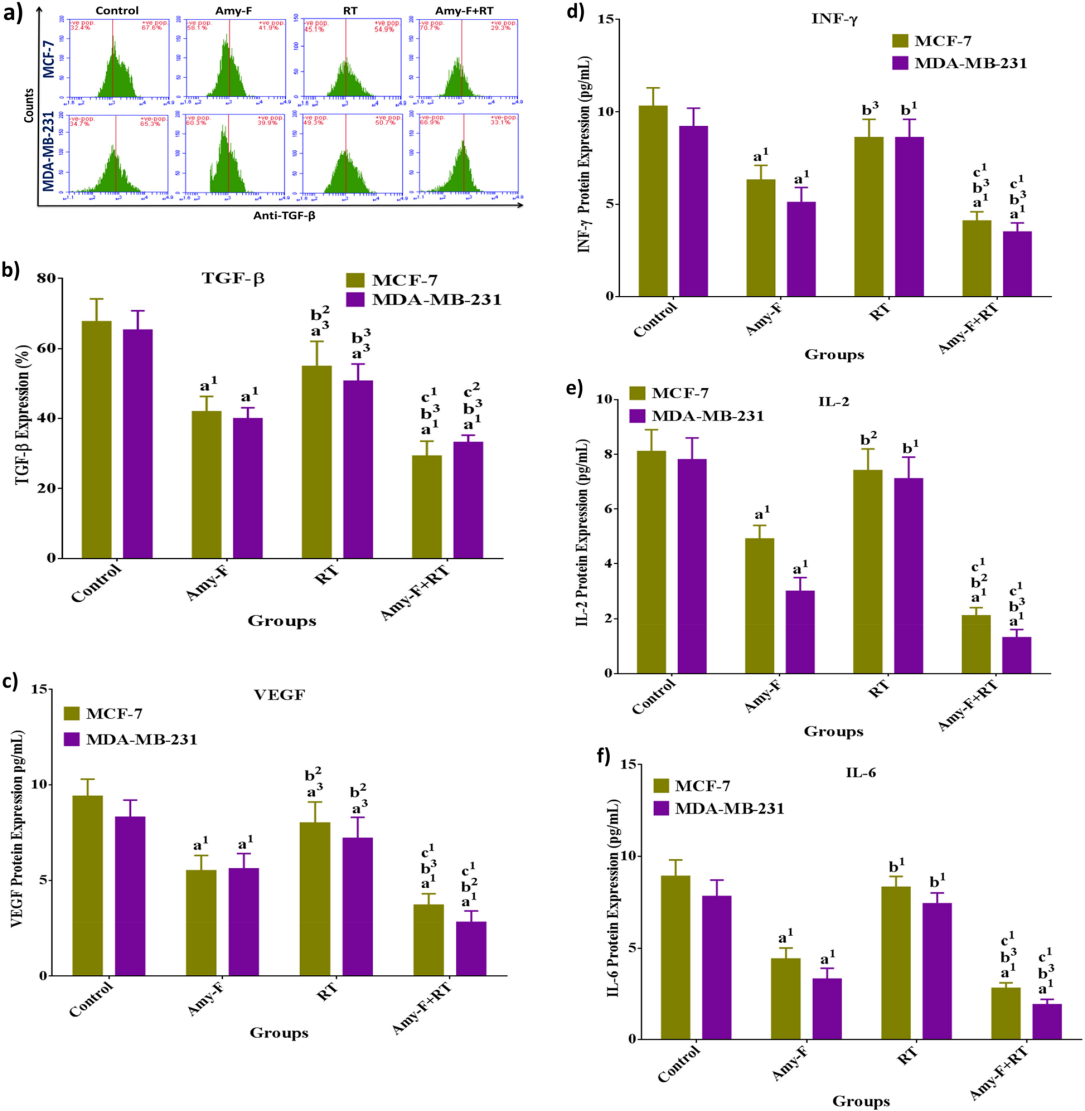
Expression of TGF-β, VEGF, INF-g, IL-2, and IL-6 in BC cells upon treatment with Amy-F and/or exposed to 4 Gy γ-irradiation. **a**, **b** % change in the expression of TGF-β in Amy-F and/or RT treated MCF-7 and MDA-MB-231 cells, respectively; **c** Fold change in the expression ratio of VEGF in Amy-F and/or RT treated MCF-7 and MDA-MB-231 cells, respectively; **d** % change in the expression of INF-g in Amy-F and/or RT treated MCF-7 and MDA-MB-231 cells, respectively; **e** Fold change in the expression ratio of IL-2 in Amy-F and/or RT treated MCF-7 and MDA-MB-231 cells, respectively; **f** Fold change in the expression ratio of IL-6 in Amy-F and/or RT treated MCF-7 and MDA-MB-231 cells, respectively. Data are mean ± SEM, a1p < 0.001, a2p < 0.01, a3p < 0.05 vs. Control group; b1p < 0.001, b2p < 0.01, b3p < 0.05 vs. Amy-F group; c1p < 0.001, c2p < 0.01, c3p < 0.05 vs. RT group

The crosstalk between TGF-β and VEGF as well as INF-γ, IL-2, and IL-6 protein levels was examined to gain insight into the underlying mechanism by which Amy-F nanoparticle and/or RT alone or in combination can modify the pro-angiogenic and metastatic capacity of BC cells. The data elucidated in (Fig. 6a-f) showed that Amy-F markedly down-regulated TGF-β protein expression by 38.1 and 38.9% in MCF-7 cells and MDA-MB-231 cells, respectively, paralleled by a significant reduction in VEGF, INF-γ, IL-2, and IL-6 protein levels by (MCF-7: 41.5, 38.8, 39.5, and 50.5%, respectively, and MDA: 32.5, 44.6, 61.5, and 57.7%, respectively) compared to control. Similarly, but to a lesser extent, MCF-7 and MDA-MB-231 cells that are exposed to 4 Gy (RT) alone exhibited a reduction in TGF-β and VEGF protein expression (MCF-7: 18.8 and 14.9%, respectively, and MDA: 22.4 and 13.3%, respectively) compared to the control cells. Whereas, breast cancer cells exposed to RT alone displayed an augmentation in TGF-β, VEGF, INF-γ, IL-2, and IL-6 levels by 31, 45.5, 36.5, 51, and 88.6%, respectively, for MCF-7 cells, and by 27.1, 28.5, 68.6, 136, and 124.2%, respectively, for MDA cells when compared with Amy-F group.

Surprisingly, when BC cells were challenged by Amy-F before RT a remarkable suppression in TGF-β protein expression (56.6%) was accompanied by a much more reduction in VEGF, INF-γ, IL-2, and IL-6 levels by 60.6, 60.2, 74.1 and 68.5%, respectively, was observed in MCF-7 cells, whereas a comparable impact was detected in MDA-MB-231 cells as indicated by reduced TGF-β expression (49.3%) as well as a significant decrease in VEGF, INF-γ, IL-2, and IL-6 levels by 66.3, 61.9, 83.3 and 75.6%, respectively, compared to control. Overall, the combination of Amy-F with RT robustly regulated the TGF-β signaling via the down-regulation of VEGF, INF-γ, IL-2, and IL-6 levels in treated BCCs and exhibited superior influence when compared to control, Amy-F, and RT groups, and thus abolished the pro-angiogenic and metastatic ability of human breast cancer cells.

## Discussion

In the present study, we aimed to suppress cell growth and proliferation of two different BC cell lines, MCF-7 and MDA-MB-231, by using a novel nanoparticle formulation as a potential inhibitor for tumor promoting factors and immunosuppressors. In particular, we have synthesized folate receptor-targeted functional drug-loaded nanoparticles. The nanoformulation consists of folic acid conjugated to *amygdalin* (Amy-F) with potential chemotherapeutic and radiosensitizing properties for effective breast cancer therapy. Specifically, the structure of Amy-F with its surface FA moiety represents the targeting machinery towards folate receptor overexpressed on BCCs. The Amy-F nanocomposite’s novel formula provided numerous characteristics such as; enhanced cytotoxicity and radio-sensitization, and controlled release of targeted drug Amy-F in response to acidic tumor medium. Ultimately, FA molecules on the Amy-F surface help in targeting the overexpressed FR-α receptors in BC cells, allowing the site-specific targeted therapy.

The effectiveness of *amygdalin* NPs were optimized in our study using a more effective pegylation approach with a functionalized nanoparticle, as contrasted to the nanoparticles used in previous studies [31]. This method facilitates the functionalization with FR-targeting ligands [32].

According to our findings, Amy-F exhibited cytotoxic capability against MCF-7 and MDA-MB-231, as revealed by the MTT assay and is devoid of apparent cytotoxicity towards normal epithelial cells (MCF-10A), suggesting the optimum drug targeting. This successful drug-selective cellular uptake might be attributed to the cell-surface annexe incorporated into the Amy-F outer shell. Cell surface-specific markers have been broadly used in cancer-targeted therapies [33]. FR-α receptors, for example, have been studied for selective nanoparticle drug delivery to BC sites [11, 12]. Selective cellular uptake was used in this study to calculate Amy-F penetrated BC cells. Amy-F-treated cells uptake fluorescent signals much more strongly than control cells, implying selective effects from FA-mediated endocytosis. As a result, dual-receptor-mediated synergic internalization notably enhanced drug system selectivity and targeted efficiency to BC cells. Furthermore, Amy-F cellular uptake was drastically reduced in normal cells, indicating FR-α dependent uptake, allowing for higher intracellular drug concentrations in cancer cells overexpressing FR-α receptors. Our findings are in agreement with a previous study that found that drug release under acidic conditions augmented the cytotoxic effects of drugs on cancer cells [34]. Noteworthy, the nanoparticle encapsulation system maintains structural integrity at pH 1.5 and the contents can only be released slowly at pH 6, inferring that the encapsulation system is stable under simulated stomach conditions and slowly releases the contents in the intestinal tract [35] which permits the administration of Amy-F nanocomposite via different routes. Simultaneously, the drug release kinetics of Amy-F in acidic (pH 6) conditions revealed the feasibility of the Amy-F formulation in cytoplasmic drug release after FR-α receptor-mediated endocytosis in BC cells. As a result, our quantitative and qualitative data show that the Amy-F system specifically and selectively binds to overexpressed FR-α receptors in cancer cells via FR- α receptor-mediated endocytosis and the responsive release within the acidic medium.

While radiosensitization of tumor cells is regarded as a promising cancer treatment approach, it is also critical to reduce the toxic effects of RT on healthy tissues surrounding tumors. Subsequently, drug/RT combinations may improve tumor control even in radio-resistant breast cancer. The main effect of RT is the production of ROS, which damages DNA and results in apoptosis. Essentially, ROS production elevation plays a role in cancer progressions, which are associated with cancer initiation and development through different signaling pathways (PI3/Akt/mTOR, PTEN, MAPK, VEGF/VEGFR, and MMPs). However, a paradox in biological systems is that ROS elevation can induce apoptotic cell death, which is an important approach in cancer therapeutics. ROS disrupts the mitochondrial membrane and opens the mitochondrial permeability transition pore, thus interfering with the mitochondrial electron transfer chain and inducing the release of Cytochrome-c leading to the activation of caspases [36].

The anticancer effect of Amy-F with RT was revealed by the reduction of BCCs viability through cell cycle arrest at G2/M and pre-G1 phases and the generation of apoptotic cell death compared to untreated cancer cell lines. Additionally, this could be attributed to the modulatory effects that are displayed on TPF, IS and signals mediators as revealed in the current study. Amy-F induced anticancer effect through *amygdalin* that has an inhibitory effect on MAPK-P38 pathways, NO, Fe, and ROS pathways as well as decreased the levels of CD4, CD80, TGF-β, VEGF, INF-g, IL-2, IL-6, and BCl-2, while increased the levels of CD8, NKG2D, and caspase-3 and finally cell cycle arrest at G/M [9, 10, 17]. This inhibitory effect of Amy-F on the tumor promoting factors could reduce the immunosuppressors and cell proliferation signaling progress in BCCs. In the same context, Lee and Moon et al. [37] reported that *amygdalin* exerted cytotoxic activities on estrogen receptors (ER)-positive MCF-7 cells, MDA-MB-231, and Hs578T TNBC cells. This could be accomplished by inhibiting Bcl-2, activating caspase-3, as well as activating proliferation signaling molecules p38 and MAPK. Furthermore, a previous study [38] reported that *amygdalin* could impede the growth of renal cell sarcoma by disrupting adhesion and migration through an integrin-dependent mechanism. Moreover, cell cycle arrest at the G2/M phase was associated with apoptosis induction, which is consistence with the findings of previous work [39].

Furthermore, the data obtained pointed to a marked reduction in TGF-β protein expression as well as enhancement of VEGF protein expression along with the abolished INF-g/IL-2/IL-6 signaling upon challenging the BC cells with Amy-F and/or RT exposure. The TGF-β is a key regulator of VEGF, IL2 and IL-6 via the inhibition of cytokines-dependent INF-g [40]. As observed in our results, the reduced levels of TGF-β could result in an induction of the inhibitory VEGF, IL2, IL-6, and INF-g expression. According to Schröder et al*.,* and Abdel-Rafei et al., [40, 41] TGF-s initiate their cellular functions by binding to the cell surface TGF-receptor complex, which activates the intracellular signaling molecules [42]. Amy-F and/or RT appeared to block INF-g/IL-2/IL-6 signaling, resulting in the induction of VEGF, which inhibits the TGF-β, a cytokine that regulates cellular processes such as cell growth, differentiation, and apoptosis.

In addition, the data obtained revealed that VEGF levels are markedly down-regulated in BC cells challenged by Amy-F and/or RT contrary to their overexpression in untreated cells. VEGF and its receptor tyrosine kinase (VEGFR) are the best-known angiogenic factors involved in angiogenesis, chemotaxis, stimulating proliferation and survival as well as the permeability of endothelial cells [43]. A variety of growth signaling pathways regulate angiogenesis. Herein, the reduction in VGEF level might be ascribed to the compromised MAPK/P38 signaling and hampered levels of CD4/CD80/IL-6/IL-2/INF-g immunosuppressors signaling in Amy-F and RT-treated cells as observed in the current study. VEGFR activation by VEGF triggers MAPK/P38 signaling, which subsequently activates through tumor promoting factors and immunosuppressors (CD4_high_/CD8_low_/CD80_high_/NKG2D^low^/IL-2_high_/IL-6_high_/INF-g_high_) [17, 41, 44, 45] identified that MAPK/P38 are essential co-transcriptional activators in endothelial cells and MAPK/P38 activity is controlled by VEGF during developmental angiogenesis. At this end, MAPK/P38 signaling and VEGF operate in a synergistic feed-forward manner to promote oncogenesis, which necessitates their targeting. In this study, Amy-F and/or RT treatment regulated efficiently this oncogenic hub. In agreement with our findings, a previous study [41] attributed the antitumor activity exhibited by *amygdalin* against colon cancer to its ability to inhibit TGF-β secretion. TGF-β could be activated through TPF and IS signaling and it promotes its nuclear translocation in hypoxic tumors [41, 44, 45] Moreover, *amygdalin* showed an anticancer effect in an in vivo model of Ehrlich solid carcinoma and was shown to reduce VEGF expression in this model and was suggested by Abdel-Rafei et al., [41] as a potential therapy for breast cancer. Hence, *amygdalin* exerted a dual targeting mechanism for MAPK/P38 and VEGF oncogenic effects. Despite the controversial data observed between low and high doses of γ-radiation in affecting angiogenesis [17, 41, 46, 47], our results are consistent with those reported that γ- radiation at a dose range of 4 Gy induced anti-angiogenic effect and reduced VEGF levels [48]. Furthermore, Abdel-Rafei et al., [41] established that 1251 seed irradiation-induced apoptosis and inhibited angiogenesis through decreasing hypoxia and VEGF expression in lung cancer.

## Conclusions

**Fig. 7:**
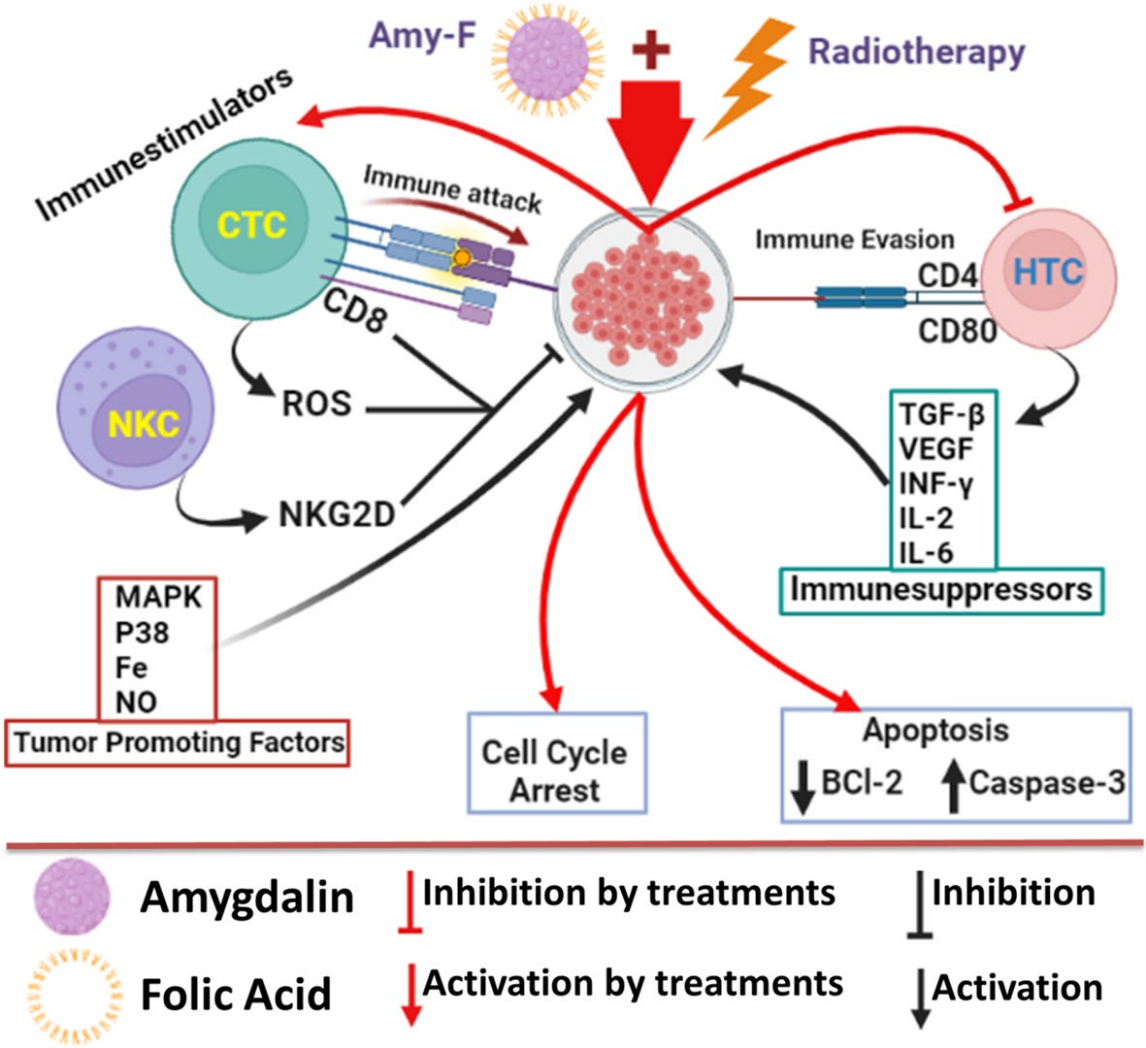
Schematic representing the mode of action of targeted functional nanoparticles (Amy-F) and RT. The Amy-F is a chemotherapy drug targeted to cancer cell surfaces via cancer cell-specific ligands. Amy-F binds to surfaces by recognizing specific receptors resulting in Amy-F internalization via endocytosis. Inside the cell, Amy-F undergoes endosomal escape leading to cytotoxic drug release. Treatment with Amy-F alone or as a radio-sensitizer (prior to RT) induces cancer cell death through modulating the tumor promoting factors and immunosuppressors of BCs by modulation of MAPK_low_, P38_low_, Fe_low_, NO_low_, ROS_high_, CD4_low_, CD8_high_, CD80_low_, NKG2D_high_, TGF-β_low_, VEGF_low_, INF-γ_low_, IL-2l_ow_, and IL-6_low_ expression. ( →) Indicates pathway direction and (T) indicates blocking functions

Overall, according to the obtained results, we could suggest that Amy-F nanoparticles could ideally target two types of breast cancer cells (MCF-7 and MDA-MB-231) alone and could also augment the influence of RT when administered preemptively. Outer surface annex (FA) provided Amy-F with a piece of successful targeting machinery that enabled the nanocomposite to anchor into the cell membrane, facilitating its entry into the cell while in the acidic environment of cancer cells (pH 6). The physicochemical characteristics of NPs, including stability, selectivity, cellular uptake, and responsive release to pH as well as anticancer efficiency, were examined thoroughly. The FA-functionalized Amy-F alone or prior RT abrogated proliferation, induced cell cycle arrest at pre-G1 and G2/M, and increased apoptosis as revealed by lower BCl-2 levels and higher caspase-3 levels of breast cancer cells. Cell-based assays revealed that Amy-F and/or RT reduced the tumor promoting factors and immunosuppressors of BCs by modulation of MAPK_low_, P38_low_, Fe_low_, NO_low_, ROS_high_, CD4_low_, CD8_high_, CD80_low_, NKG2D_high_, TGF-β_low_, VEGF_low_, INF-γ_low_, IL-2l_ow_, and IL-6_low_ expression as shown in (Fig. 7). The exposure of both breast cancer cells to Amy-F before RT enhanced their response and augmented radiosensitivity, suggesting the emerging role of Amy-F nanoparticle as a potential radiosensitizer in therapeutic regimens for the treatment of breast cancer cells. From the future perspective of the work, since the targeted nanomedicines have tremendous therapeutic potential in BC treatment over their free drug counterparts. Thus, the Amy-F nanocomposite has significant capabilities to interrupt and break off breast cancer cell proliferation by tumor-promoting factors/ immunosuppressive modulators, and potentiating the effects of gamma radiation. It could be recommended to use Amy-F nanocomposite as a potential radiosensitizer in BC therapeutic regimens in vivo, modified it by adding the iron oxide nano to the composite and evaluating the promising effects by imaging assays as western blot, fluorescent microscope, and ICP-mass.

## Data Availability

All data generated or analyzed during this study are included in this manuscript.

## References

[1] Wu P (2019). Enhanced anti-tumor efficacy of hyaluronic acid modified nanocomposites combined with sonochemotherapy against subcutaneous and metastatic breast tumors. Nanoscale.

[2] Arnold M (2022). Current and future burden of breast cancer: Global statistics for 2020 and 2040. Breast.

[3] Saad AM, Hussein MA, El-Afandey AM (2022). Perception of Female Health Care Providers Regarding Breast Cancer at El Minia Oncology Center. Egyp J Hosp Med.

[4] Bai D-P (2017). Zinc oxide nanoparticles induce apoptosis and autophagy in human ovarian cancer cells. Int J Nanomed.

[5] Shi R, Tang YQ, Miao H (2020). Metabolism in tumor microenvironment: Implications for cancer immunotherapy. MedComm.

[6] Han X (2014). Tissue factor in tumor microenvironment: a systematic review. J Hematol.

[7] Sullivan SP, Murthy N, Prausnitz MR (2008). Minimally invasive protein delivery with rapidly dissolving polymer microneedles. Adv Mater.

[8] Torchilin VP (2014). Multifunctional, stimuli-sensitive nanoparticulate systems for drug delivery. Nat Rev Drug Discovery.

[9] Liczbiński P, Bukowska B (2018). Molecular mechanism of amygdalin action in vitro: review of the latest research. Immunopharmacol Immunotoxicol.

[10] El-Desouky MA (2020). Anticancer Effect of Amygdalin (Vitamin B-17) on Hepatocellular Carcinoma Cell Line (HepG2) in the Presence and Absence of Zinc. Anticancer Agents Med Chem.

[11] Liu M (2019). Novel multifunctional triple folic acid, biotin and CD44 targeting pH-sensitive nano-actiniaes for breast cancer combinational therapy. Drug Delivery.

[12] Jurczyk M (2021). Single-versus dual-targeted nanoparticles with folic acid and biotin for anticancer drug delivery. Pharmaceutics.

[13] Golombek SK (2018). Tumor targeting via EPR: Strategies to enhance patient responses. Adv Drug Deliv Rev.

[14] Askar MA (2021). Dual Hyaluronic Acid and Folic Acid Targeting pH-Sensitive Multifunctional 2DG@ DCA@ MgO-Nano-Core–Shell-Radiosensitizer for Breast Cancer Therapy. Cancers.

[15] Van de Loosdrecht A (1994). A tetrazolium-based colorimetric MTT assay to quantitate human monocyte mediated cytotoxicity against leukemic cells from cell lines and patients with acute myeloid leukemia. J Immunol Methods.

[16] Baibarac M (2019). Optical properties of folic acid in phosphate buffer solutions: the influence of pH and UV irradiation on the UV-VIS absorption spectra and photoluminescence. Sci Rep.

[17] Thabet NM (2022). Multifunctional nanocomposites DDMplusAF inhibit the proliferation and enhance the radiotherapy of breast cancer cells via modulating tumor-promoting factors and metabolic reprogramming. Cancer Nanotechnol.

[18] Livak KJ, Schmittgen TD (2001). Analysis of relative gene expression data using real-time quantitative PCR and the 2(-Delta Delta C(T)) Method. Methods.

[19] Ruman U (2021). Synthesis and characterization of chitosan-based nanodelivery systems to enhance the anticancer effect of sorafenib drug in hepatocellular carcinoma and colorectal adenocarcinoma cells. Nanomaterials.

[20] Marian E (2020). Synthesis, characterization of inclusion compounds of amygdalin with β-cyclodextrin and sod-like activity and cytotoxicity on hela tumor cells. Arab J Chem.

[21] Zipare K, Bandgar S, Shahane G (2018). Effect of Dy-substitution on structural and magnetic properties of MnZn ferrite nanoparticles. J Rare Earths.

[22] He Y (2009). Complexation of anthracene with folic acid studied by FTIR and UV spectroscopies. Spectrochim Acta A Mol Biomol Spectrosc.

[23] Thakur A (2019). Physicochemical properties, mineral composition, FTIR spectra and scanning electron microscopy of wild apricot kernel press cake. Int J Food Sci Nutr.

[24] Nasser HM (2021). Effect of sorafenib on liver biochemistry prior to vitamin b17 coadministration in ehrlich ascites carcinoma mice model: preliminary phase study. Biochemistry Letters.

[25] Garg UK (2007). Removal of hexavalent chromium from aqueous solution by agricultural waste biomass. J Hazard Mater.

[26] Mohammed E (2014). Qualitative and quantitative determination of folic acid in tablets by FTIR spectroscopy. IJAPBC.

[27] Jaszczak-Wilke E (2021). Amygdalin: toxicity, anticancer activity and analytical procedures for its determination in plant seeds. Molecules.

[28] El-Batal AI (2022). Antimicrobial synergism and antibiofilm activity of amoxicillin loaded citric acid-magnesium ferrite nanocomposite: Effect of UV-illumination, and membrane leakage reaction mechanism. Microb Pathog.

[29] Uppuluri S (2000). Core–shell tecto (dendrimers): I. Synthesis and characterization of saturated shell models. Adv Mater.

[30] Bonn M, Hunger J (2021). Between a hydrogen and a covalent bond. Science.

[31] Ivask A (2015). Toxicity of 11 metal oxide nanoparticles to three mammalian cell types in vitro. Curr Top Med Chem.

[32] Bajracharya R (2022). Functional ligands for improving anticancer drug therapy: current status and applications to drug delivery systems. Drug Deliv.

[33] Wang Z (2018). CD44 directed nanomicellar payload delivery platform for selective anticancer effect and tumor specific imaging of triple negative breast cancer. Nanomedicine.

[34] Guo Y (2017). Hyaluronic acid and Arg-Gly-Asp peptide modified graphene oxide with dual receptor-targeting function for cancer therapy. J Biomater Appl.

[35] Zhuo S (2020). pH-sensitive biomaterials for drug delivery. Molecules.

[36] Aggarwal V (2019). Role of reactive oxygen species in cancer progression: molecular mechanisms and recent advancements. Biomolecules.

[37] Lee HM, Moon A (2016). Amygdalin regulates apoptosis and adhesion in Hs578T triple-negative breast cancer cells. Biomolecules.

[38] Juengel E (2016). Amygdalin blocks the in vitro adhesion and invasion of renal cell carcinoma cells by an integrin-dependent mechanism. Int J Mol Med.

[39] Li Z (2016). Isoalantolactone induces apoptosis in human breast cancer cells via ROS-mediated mitochondrial pathway and downregulation of SIRT1. Arch Pharmacal Res.

[40] Schröder M (2003). Different modes of IL-10 and TGF-β to inhibit cytokine-dependent IFN-γ production: consequences for reversal of lipopolysaccharide desensitization. J Immunol.

[41] Abdel-Rafei MK, Askar MA, Azab KS, El-Sayyad GS, El Kodous MA, El Fatih NM (2023). FA-HA-Amygdalin@Fe2O3 and/or γ-rays affecting SIRT1 regulation of YAP/TAZ-p53 signaling and modulates tumorigenicity of MDA-MB231 or MCF-7 cancer cells. Curr Cancer Drug Targets.

[42] Massagué J (2012). TGFβ signalling in context. Nat Rev Mol Cell Biol.

[43] Sakabe M (2017). YAP/TAZ-CDC42 signaling regulates vascular tip cell migration. Proc Natl Acad Sci.

[44] Zhang J (2016). Macrophage migration inhibitory factor regulating the expression of VEGF-C through MAPK signal pathways in breast cancer MCF-7 cell. World J Surg Oncol.

[45] Retecki K (2021). The immune landscape of breast cancer: strategies for overcoming immunotherapy resistance. Cancers.

[46] Hlushchuk R (2008). Tumor recovery by angiogenic switch from sprouting to intussusceptive angiogenesis after treatment with PTK787/ZK222584 or ionizing radiation. Am J Pathol.

[47] Vincenti S (2011). HUVEC respond to radiation by inducing the expression of pro-angiogenic microRNAs. Radiat Res.

[48] Park M-T (2012). Radio-sensitivities and angiogenic signaling pathways of irradiated normal endothelial cells derived from diverse human organs. J Radiat Res.

